# Assessment of burnout in medical undergraduate students in Riyadh, Saudi Arabia

**DOI:** 10.1186/s12909-019-1468-3

**Published:** 2019-01-25

**Authors:** Youssef Altannir, Wedad Alnajjar, Syed Osama Ahmad, Mustafa Altannir, Fouad Yousuf, Akef Obeidat, Mohamad Al-Tannir

**Affiliations:** 1 College of Medicine, Alfaisal University, P.O. Box 50927, Takhasusi Road, Riyadh, 11533 Saudi Arabia; 2 Clinical & Applied Research Department, Research Center, King Fahad Medical City, P.O. Box 59046, Riyadh, 11525 Saudi Arabia

**Keywords:** Burnout, Medical students, Anxiety, Depression, Depersonalization

## Abstract

**Background:**

To assess the prevalence of burnout symptoms among preclinical and clinical medical students studying at AlFaisal University in Riyadh, Saudi Arabia.

**Methods:**

A cross-sectional study was conducted using Maslach Burnout Inventory questionnaire on 276 medical students from Alfaisal University, Riyadh, Saudi Arabia. The study was approved by Alfaisal University research ethics committee. Chi-square test was used to identify statistically significant differences, and binary logistic regression was used to identify predictors of burnout.

**Results:**

276 entered into final data analysis with a mean age 20.62 ± 1.58, of whom 54% were males, and 46% were females. The overall burnout prevalence was 13.4%, of which PA was the most prevalent domain of burnout with 64.9%. Female gender was a significant predictor of EE and DP [OR = 4.34; 95% Cl 1.86–10.13; *P*-value 0.001] and [OR = 2.01; 95% Cl 1.07–3.79; P-value 0.030] respectively as per multivariate analysis for demographic characteristics. Regarding the total level of burnout, females (75.7%) had significantly higher levels of burnout compared to males (41.4%); (*P*-value < 0.001).

**Conclusion:**

Burnout is prevalent among medical student. Gender was found to exhibits effect on the burnout. Mutual proactive strategies and reactive coping mechanisms between the students and the universities are encouraged to prevent and reduce burnout among medical students.

## Background

Medical schools aim to graduate professional physicians equipped and trained with the knowledge, competency to promote the nation health and well-being, and to advance medical science. Besides, medical colleges make every effort to support medical students during their study. Medical schools carry out robust selection procedures to recognize altruistic and intelligent students with a resilient obligation to abovementioned goals [1].

Medical undergraduate training is lengthy and emotionally demanding. Several studies have shown high levels of psychological problems in medical students at different points of their training and academic life [2, 3]. Hence, Medical students are always vulnerable to burnout syndrome due to psychosocial stressors throughout the academic and training life [4]. Burnout is a syndrome of emotional exhaustion, cynicism, and low professional efficacy that frequently occurs among individuals who do ‘people work’ of some kind [5]. The term is useful to reflect on peoples who participate in activities that are mentally and psychologically similar to work, like students [6, 7].

Previous literature reported worsened mental health after medical students begin school and continue so during their training and after their graduation [8]. Medical school is a stressful learning environment since students are always expected to learn and memorize an enormous sum of information within a limited amount of time [9–11]. Students also face financial stressors such as having to secure scholarships, repaying or managing student debt loans, amongst other things which further increases the burden of work demands. Moreover, a dearth of time for relaxation and leisure, family and friends, preparation for the residency program, the choice of a specialty and the late monthly income also exacerbate stress among medical students [4, 12]. As a result, stress, burnout, and sleep disorders (such as insomnia) gradually develop throughout the academic years [9, 11, 13]. All of these are interconnected problems which influence each other and could lead to serious health consequences such as “anxiety disorders, depression, substance abuse, suicidal ideation, thoughts of dropping out, reduced empathy, low motivation for learning, and low academic conduct” [11, 14]. Studies have also shown that medical students have a poor mental quality of life when compared to individuals of the same age in the general public [13].

A varied range of burnout levels among medical students have been reported worldwide. Recent studies indicated a high prevalence of burnout is reaching 71% and up to 76.8% [9, 15, 16]. However, other studies showed lesser levels of burnout ranging between 10 and 55% [1, 13, 17–20]. Researches from Saudi Arabia have identified a high prevalence of burnout among medical students [13, 21–23]. Generally, the prevalence of burnout is considerably dissimilar across countries. The Middle East and Oceania countries have a higher prevalence of burnout than other countries in other continents [24]. The varied levels and differences of burnout may perhaps be explained by the diversity of instruments used to measure burnout levels or due to the different social and circumstantial determining factors of burnout.

Although various literature has been reporting burnout among health care professionals, including, physicians and nurses [25–28], the in-depth literature review showed a scarcity in the epidemiological studies exploring the prevalence and impact of burnout among medical students per se in Saudi Arabia [21, 23]. This study aimed to assess the prevalence of burnout symptoms among preclinical and clinical medical students studying at AlFaisal University in Riyadh, Saudi Arabia. Hence, this study will be an added value to the Saudi literature that may encourage and enable the scientific community to early detect burnout syndrome and to adopt proactive and preventive measures.

## Methods

### Study design and setting

A cross-sectional study was conducted between January and February 2016 at the College of Medicine at AlFaisal University in Riyadh, Saudi Arabia. AlFaisal University is a private non-profit institute set up under the patronage of King Faisal Foundation. The college of medicine inducted its first batch in 2008. The medical program comprises of a 6-year MBBS degree. The preclinical phase comprises of several basic science courses delivered during the first three years of the program. The clinical phase comprises of fourth and fifth years, where students rotate within different hospital departments for 9 weeks each. The Internship is considered as the sixth year. The student body within AlFaisal is composed of various ethnic and socioeconomic backgrounds.

### Participants, sampling method, and sample size

Using a stratified convenient sampling technique, we recruited medical students from the preclinical and clinical years. For preclinical students, the surveys were distributed on campus following lectures and during break hours by the authors. Whereas, for clinical students, the surveys were distributed in King Faisal Specialist Hospital and Research Center and Security Forces Hospital. A total of 276 participants volunteered to fill the paper-based questionnaire out of 400 students approached based on stratification per academic year; first year (*n* = 87), second year (*n* = 77), third year (*n* = 66), fourth year (*n* = 28), and fifth year (*n* = 18) undergraduate medical students. The sample size of 278 medical students was determined valid by Rao Soft® sample size calculator at 80% power and 5% margin of error.

### Ethical considerations

Ethical approval was obtained from the institutional review board at AlFaisal University under exempt approval category. Study information sheet was attached to the questionnaire indicating that students’ participation is voluntary and the identity will not be collected so their information can never be matched to their responses. Verbal consent was obtained from each student before filling out the survey as granted by the institutional review board.

### Data collection instrument and procedure

The medical students were asked to fill a structured questionnaire that has been designed and formulated based on the information provided by the Maslach Burnout Inventory (MBI) [29], which calculates burnout score using 22-items for three categories of burnout symptoms “EE, DP, PA” [30, 31]. Additionally, the questionnaire has been modified to include demographic data (age, gender, and school year) and questions related to the target population.

The MBI questionnaire was formulated to assess whether a person is at risk of burnout [30–32]. The MBI Questionnaire is a 22-item instrument that measures three sections: “EE,” “DP,” “PA.” EE is the feeling of being completely drained, both emotionally and physically, due to extreme overwork [9]. DP is a combination of a negative, skeptical behavior, and a feeling of indifference towards others. Low PA is the propensity to judge oneself badly or unfavorably especially towards one’s own work [32, 33]. EE was measured using seven items (For example: I am at the edge of breaking down due to the responsibilities I have). DP was measured using seven items (For example I feel I look at my colleagues as an object, as a person with no personality or feelings). PA was measured using eight items (For example I accomplish many worthwhile things during the day while attending AlFaisal University, College of Medicine). All survey items were scored on a scale from 0 to 6; (0 = Never, 1 = Few times per year, 2 = Once a month, 3 = Few times per month, 4 = Once a week, 5 = Few times per week, 6 = Everyday). DP and EE are inversely proportional to PA but directly proportional to burnout [33].

MBI assesses a person’s risk of burnout but doesn’t provide a diagnosis. For a definitive diagnosis, a clinical assessment is required alongside MBI [32]. High levels of burnout are represented via high scores of EE and DP and a low score of PA [9]. Moreover, the survey was modified to include demographic data (age, gender and medical school year).

### Statistical analysis

Data analysis was conducted using IBM SPSS version 22. All categorical variables such as age, gender, and medical school year were presented as numbers and percentages. Chi-square and Fisher’s exact tests were used interchangeably, and these tests helped determine the significant association between categorical variables. Backward multiple logistics regression analysis was conducted for each of the three MBI component levels to examine the relationship between burnout and general characteristics of the participants. The odds ratios (OR) and 95% confidence intervals (95% CI) were also calculated. A *p*-value of less than 0.05 was considered to be of statistical significance.

## Results

A total of 276 students participated in the study with a mean age 20.62 ± 1.58, of whom 149 (54%) were male. Slightly more than Two-Thirds were between the age group (19–21). About 230 (83.4%) were preclinical, and 46 (16.6%) were clinical medical undergraduates Table 1.

**Table 1:** Demographic characteristics of the participants (*n* = 276)

Characteristics	Categories	n (%)
Age	16–18	14 (5.1)
	19–21	190 (68.8)
	> 21	72 (26.1)
	*mean ± SD*	*20.62 ± 1.58*
Gender	Male	149 (54)
	Female	127 (46)
School Year	First	87 (31.5)
	Second	77 (27.9)
	Third	66 (23.9)
	Fourth	28 (10.1)
	Fifth	18 (6.5)

About one-third of the students reported moderate level of EE and 17,4% reported moderate-high levels; however, the students EE mean subscale score was 18.53 ± 10.25 indicating moderate levels. The majority of the students 157 (56.9%) reported a higher level of DP. The DP mean subscale score was 14.20 ± 9.22 indicating high level. Moreover, about 14.9% of the students showed low levels of PA and 20.3% showed moderate PA levels Table 2.

**Table 2:** Mean scores and levels of MBI subscales

Characteristics	mean subscales score	n (%)
Emotional Exhaustion	18.53 ± 10.25	
≤ 17 (Low-Level)		138 (50)
18–29 (Moderate-Level)		90 (32.6)
≥30 (High-Level)		48 (17.4)
Depersonalization	14.20 ± 9.22	
≤ 5 (Low-Level)		55 (19.9)
6–11 (Moderate-Level)		64 (23.2)
≥12 (High-Level)		157 (56.9)
Personal Accomplishment	28.73 ± 9.51	
≤ 33 (High-Level)		179 (64.9)
34–39 (Moderate-Level)		56 (20.3)
≥40 (Low-Level)		41 (14.9)

Table 3 summarizes the distribution of burnout level according to participants’ demographics. The majority of the students who indicated high EE were among the age group 19–21 year. 34 (70.8%) females reported higher levels of EE than males 14 (29.2%). The levels of EE were found decreasing as the school year progress.

**Table 3:** Comparison of burnout levels with students’ demographics

		Emotional Exhaustion			Depersonalization			Personal Accomplishment		
		≤ 17	18–29	≥ 30	≤ 5	6–11	≥12	≤ 33	34–39	≥40
		(Low)	(Moderate)	(High)	(Low)	(Moderate)	(High)	(High)	(Moderate)	(Low)
Age	16–18	6 (4.3%)	3 (3.3%)	5 (10.4%)	1 (1.8%)	3 (4.7%)	10 (6.4%)	9 (5.0%)	5 (8.9%)	0
	18–21	91 (65.9%)	68 (75.6%)	31 (64.6%)	39 (70.9%)	42 (65.6%)	109 (69.4%)	126 (70.4%)	38 (67.9%)	26 (63.4%)
	> 21	41 (29.7%)	19 (21.1%)	12 (25.0%)	15 (27.3%)	19 (29.7%)	38 (24.2%)	44 (24.6%)	13 (23.2%)	15 (36.6%)
Gender	Male	91 (65.9%)	44 (48.9%)	14 (29.2%)	35 (63.6%)	41 (64.1%)	73 (46.5%)	88 (49.2%)	34 (60.7%)	27 (65.9%)
	Female	47 (34.1%)	46 (51.1%)	34 (70.8%)	20 (36.4%)	23 (35.9%)	84 (53.5%)	91 (50.8%)	22 (39.3%)	14 (34.1%)
School year	First	35 (25.4%)	30 (33.3%)	22 (45.8%)	16 (29.1%)	17 (26.6%)	54 (34.4%)	51 (28.5%)	22 (39.3%)	14 (34.1%)
	Second	38 (27.5%)	28 (31.1%)	11 (22.9%)	16 (29.1%)	18 (28.1%)	43 (27.4%)	59 (33.0%)	10 (17.9%)	8 (19.5%)
	Third	30 (21.7%)	23 (25.6%)	13 (27.1%)	6 (10.9%)	16 (25.0%)	44 (28.0%)	51 (28.5%)	10 (17.9%)	5 (12.2%)
	Fourth	21 (15.2%)	7 (7.8%)	0	12 (21.8%)	8 (12.5%)	8 (5.1%)	9 (5.0%)	11 (19.6%)	8 (19.5%)
	Fifth	14 (10.1%)	2 (2.2%)	2 (4.2%)	5 (9.1%)	5 (7.8%)	8 (5.1%)	9 (5.0%)	3 (5.4%)	6 (14.6%)

Moreover, the highest percentages of the students who reported low 39 (70.9%), moderate 68 (75.6%) and high 109 (69.4%) DP were between the age 19–21. More than half of the students who reported high DP were females; on the other hand, 41 (64.1%) of the students who reported moderate DP were males. Again, the highest percentages of students who reported higher levels of DP were in the preclinical years when compared to the students who were in the clinical years. The majority of the students who reported low PA were males and between the age group 19–21, 27 (65.9%) and 26 (63.4%) respectively. The PA levels were higher among students in the preclinical years compared to students in the clinical years Table 3.

Table 4 shows the comparison of differences in the burnout levels according to students’ demographics. A significant difference was found in the EE levels and gender (*p* = < 0.001) and school year (*p* = 0.008). Moreover, a significant difference was found in the DP levels and gender (*p* = 0.016) and school year (*p* = 0.015). However, a significant difference was noted in the PA levels and school year (*p* = < 0.001).

**Table 4:** Comparison of differences in the burnout levels according to students’ demographics

		Emotional Exhaustion	Depersonalization	Personal Accomplishment
		*P*-value	*P*-value	*P*-value
Age	16–18	0.212	0.665	0.197
	18–21			
	> 21			
Gender	Male	< 0.001*	0.016*	0.081
	Female			
School year	First	0.008*	0.015*	< 0.001*
	Second			
	Third			
	Fourth			
	Fifth			

*Significant at *p*-value< 0.005

Table 5 shows the tabulation of the student’s demographics and high levels of EE, DP, and LPA. The tabulation revealed that a significant difference was found between gender and high levels of EE, DP, and PA (*p* = 0.015).

**Table 5:** Tabulation of the student’s demographics and high levels of EE, DP, and PA

		Emotional Exhaustion	Depersonalization	Personal Accomplishment	*p*- value
Age	16–18	5 (10.4%)	10 (6.4%)	9 (5.0%)	0.983
	18–21	31 (64.6%)	109 (69.4%)	126 (70.4%)	
	> 21	12 (25.0%)	38 (24.2%)	44 (24.6%)	
Gender	Male	14 (29.2%)	73 (46.5%)	88 (49.2%)	0.015*
	Female	34 (70.8%)	84 (53.5%)	91 (50.8%)	
School year	First	22 (45.8%)	54 (34.4%)	51 (28.5%)	0.710
	Second	11 (22.9%)	43 (27.4%)	59 (33.0%)	
	Third	13 (27.1%)	44 (28.0%)	51 (28.5%)	
	Fourth	0	8 (5.1%)	9 (5.0%)	
	Fifth	2 (4.2%)	8 (5.1%)	9 (5.0%)	

*Significant at *p*-value< 0.005

Female gender was a significant predictor of emotional exhaustion [OR = 4.34; 95% Cl 1.86–10.13; *P*-value 0.001] as per multivariate analysis for demographic characteristics and emotional exhaustion. Similarly, female gender was a significant predictor of depersonalization [OR = 2.01; 95% Cl 1.07–3.79; P-value 0.030] as per multiple regression analysis for demographic characteristics and depersonalization Table 6.

**Table 6:** logistic regression analysis for burnout subscales and students’ demographics

	EE			DP			PA		
Parameter	OR	*P* – value	95% C.I	OR	*P* – value	95% C.I	OR	*P* – value	95% C.I
Age	1.22	0.622	[0.553–2.696]	2.017	0.116	[0.841–4.836]	1.46	0.394	[0.611–3.498]
Gender	2.01	0.030*	[1.070–3.791]	4.338	0.001*	[1.858–10.129]	0.50	0.056	[0.247–1.019]
School Year	0.84	0.338	[0.591–1.198]	0.706	0.097	[0.467–1.066]	1.06	0.756	[0.726–1.554]

*Significant at *p*-value< 0.005

## Discussion

Utilization of the MBI scale in this cross-sectional study was acceptable and feasible to assess medical students’ burnout levels. Medical students participated in the present study revealed frequent emotional exhaustion, high levels of depolarization, and high personal accomplishment. The gender may predict medical students’ emotional exhaustion and depolarization.

Our study revealed that the level of burnout decreased as the students advanced to from pre-clinical to clinical years. The study noted a significant correlation between gender and burnout subscales. Comparing our findings with the previously reported studies in the literature could not be done appropriately, mainly due to curriculum difference between medical schools worldwide [9, 10, 13, 32, 34–42].

The present study found that a large proportion of the female medical students observed a relatively higher emotional exhaustion and depersonalization in contrast to male medical students. Whereas, female students had lower rates of personal accomplishment comparing to male medical students. This highlights the fact that females are more burnout across all the 3 domains assessing burnout. In this study, females had three times greater predisposition to burnout in comparison to the males. ALMalki et al. (2017) and showed that medical students’ gender was not a risk factor for EE, DP, and PA among medical students [21, 23]. However, a systematic review study has reported gender as a significant predictor of burnout or for one of the burnout subscales [43]. A study carried out on osteopathic medical students revealed that gender had a significant impact on burnout, mainly via influencing its three dimensions. The osteopathic female students had a relatively 1.5 times higher burnout rates than male students. They also exhibited higher rates of EE and lower rates of PA compared to males [33]. The majority of the studies agreed that the female gender was significantly more predisposed to developing high levels of stress and burnout when compared to their male counterparts. Nevertheless, the cause remains unclear [29]. Some studies are currently available in the literature; however, they provide contradictory results regarding the influence of gender on burnout. Several studies suggested that females have a higher likelihood to view challenging or threatening situations as stressful, compared to males [9, 37]. In contrast, other studies showed no association between gender and burnout [32, 41].

The explanation for the higher levels of burnout among female medical students in Saudi Arabia could be contributed to cultural, social, and religious factors and hence influence their PA and EE. The Saudi cultural norm does not permit intermingling of the sexes, which could affect their exposure in medical training [44]. Besides, Saudi females are preserving family life. Therefore, female students need to make an additional effort to reach their goals with extra physical and mental hours’ expended coping with study and family needs [45]. This could be a potential factor for the increasing the prevalence of stress and burnout among female medical students when compared to male students [45].

Burnout gradually progresses over the years of medical education. Medical students; during the preclinical and clinical periods are expected to be more responsible toward patients and expose to an extensive volume of knowledge and practice. The rates of burnout among preclinical medical students (32.4%) and clinical medical students (2.7%) are in accordance with previous studies [9, 10, 13, 46]. Feras et al., (2016) stated that there is a high level of burnout (75%) among preclinical medical students at the American University of Beirut, Lebanon. In addition, the article mentioned that female student, as well as first-year medical students, exhibited higher rates of burnout in comparison to male students and the rest of the academic years, which correlated with our study’s results [9]. Moreover, Guthrie et al. and Sreeramareddy et al. reported similar results whereby the first year medical students have higher levels of burnout in comparison to second-year medical students [37, 42]. Sreeramareddy et al. noted that second-year medical students have a lower rate of burnout as a result of the improved gradual adaptation an individual experience with time throughout the pressure living environment [42].

Our study showed that the prevalence of EE, DP, and PA is decreasing as the medical students’ progress from preclinical to clinical years and that academic year was not a risk factor for burnout. Interestingly, a study done in Spain exhibited different findings. It stated that the prevalence of burnout was significantly lower among preclinical medical students (14.8%) as compared to clinical students (37.5%) [32]. However, AlMalki et al. have reported a negative trend between the level of EE, DP, and PA compared to an academic year, and that academic year was not a predictor for burnout [21].

Our study found high levels of emotional exhaustion and depersonalization among themedical students. A study conducted by West et al. revealed an important reciprocal relation between emotional exhaustion, depersonalization, burnout and high-impact outcomes such as suicide, low personal welfare, professionalism, and commitment among medical students and physicians [29, 30]. Another study showed the negative influence burnout has on the psychological and physical welfare of preclinical medical students. It indicated that high emotional exhaustion is linked to low physical well-being [11, 14]. Burnout also seems to influence the quality of life of undergraduate medical students, which in turn affects health care yet the quantitative effect remains unknown [14, 15].

Our study aimed to measure the rate of burnout amongst preclinical and clinical medical students in Saudi Arabia. However, this study has some limitations, since it is not representative of all the national undergraduate medical student burnout level, as it measures the burnout level in only one university in the Kingdom of Saudi Arabia. Moreover, the medical students were not approached on the basis of random sampling but rather on the basis of convenience sampling, which means that students with higher levels of burnout might have been missed as they refused to fill in the survey. This might be especially true for students in clinical years, as several fourth and fifth-year students refused to fill in the survey and were difficult to approach. Moreover, since the research is a cross-sectional study, it was not able to determine the causal relationships. The participation in this study was, and participants were recruited non-randomly, therefore hosting selection bias. Although we stratified distributed the sample size equally across the preclinical and clinical years, the response rate was small among the first and second years.

### Recommendations

High levels of burnout were observed among participants. Several actions need to be implemented to reduce student burnout, as students graduating medical school with high levels of burnout have an increased likelihood of developing severe burnout during residency training, and this may lead to detrimental repercussions in regards to student careers and patient healthcare. Persistent burnout can lead to other mental and physical health care problems such as depression, drug abuse, alcoholism amongst other consequences.

Several strategies were proposed to cope and manage stressors and burnout. Strategies that encompass engagement process such as problem solving, positive reflection and expression of emotion, enable students’ adaptation [9, 47, 48] that lessens anxiety and depression and their impacts on students’ mental integrity [49] and physical well-being [48]. Involving music and physical exercise are extracurricular activities that have been linked to reduced stress and burnout levels in medical students [9].

Moreover, organizational strategies have shown significant reduction in burnout amongst medical doctors [50]. An essential component of these strategies is the continuing assessment of mental health outcomes across all four years of the curriculum. Efforts should be focused at changing the educational and clinical environments to lessen avoidable stressors and construct more optimistic environments for teaching and clinical practice [24].

## Conclusion

Considerable levels of burnout were observed among study participants especially depolarization levels. Although the prevalence of burnout levels was decreasing as the school year progress, years of school was not a risk factor for burnout. Several strategies are needed to reduce medical students’ burnout, as it may accumulate over the years and lead to detrimental repercussions in regards to student careers. Longitudinal studies are required to explore the pattern of burnout among medical students from school admission until graduation.

## Abbreviations

DP: Depersonalization
EE: Emotional Exhaustion
MBI: Maslach Burnout Inventory-Educators Survey
OR: Odds Ratios
PA: Personal Accomplishment
